# An Optimized Method to Detect BDS Satellites’ Orbit Maneuvering and Anomalies in Real-Time

**DOI:** 10.3390/s18030726

**Published:** 2018-02-28

**Authors:** Guanwen Huang, Zhiwei Qin, Qin Zhang, Le Wang, Xingyuan Yan, Xiaolei Wang

**Affiliations:** College of Geology Engineering and Geomantic, Chang’an University, 126 Yanta Road, Xi’an 710054, China; huang830928@chd.edu.cn (G.H.); 2014026004@chd.edu.cn (L.W.); 2016026025@chd.edu.cn (X.Y.); 2015026004@chd.edu.cn (X.W.)

**Keywords:** GEO/IGSO, MEO, real-time detection, orbital maneuver, orbital anomaly, median robust

## Abstract

The orbital maneuvers of Global Navigation Satellite System (GNSS) Constellations will decrease the performance and accuracy of positioning, navigation, and timing (PNT). Because satellites in the Chinese BeiDou Navigation Satellite System (BDS) are in Geostationary Orbit (GEO) and Inclined Geosynchronous Orbit (IGSO), maneuvers occur more frequently. Also, the precise start moment of the BDS satellites’ orbit maneuvering cannot be obtained by common users. This paper presented an improved real-time detecting method for BDS satellites’ orbit maneuvering and anomalies with higher timeliness and higher accuracy. The main contributions to this improvement are as follows: (1) instead of the previous two-steps method, a new one-step method with higher accuracy is proposed to determine the start moment and the pseudo random noise code (PRN) of the satellite orbit maneuvering in that time; (2) BDS Medium Earth Orbit (MEO) orbital maneuvers are firstly detected according to the proposed selection strategy for the stations; and (3) the classified non-maneuvering anomalies are detected by a new median robust method using the weak anomaly detection factor and the strong anomaly detection factor. The data from the Multi-GNSS Experiment (MGEX) in 2017 was used for experimental analysis. The experimental results and analysis showed that the start moment of orbital maneuvers and the period of non-maneuver anomalies can be determined more accurately in real-time. When orbital maneuvers and anomalies occur, the proposed method improved the data utilization for 91 and 95 min in 2017.

## 1. Introduction

BeiDou Navigation Satellite System (BDS) has been providing continuous Positioning, Navigation, and Timing (PNT) services across the whole Asia-Pacific region since 27 December 2012. It aims to serve global users upon its completion in 2020 [1,2,3,4,5,6,7,8]. The BDS is influenced by Earth’s non-spherical gravity and other perceptual factors, which lead to long-term perturbations of the offset of the satellite location and orbital elements. In order to keep the satellite in the normal range of the design orbit, orbit maneuvering is necessary to adjust the orbit of the satellite using the propulsion systems. In maintaining geosynchronous characteristics, the frequency of orbit maneuvering for Geostationary Orbit (GEO) and Inclined Geosynchronous Orbit (IGSO) satellites is higher than in Medium Earth Orbit (MEO) satellites. The position of satellite will vary by tens of kilometers because of orbit maneuvering, causing serious impact on the orbit determination and the service performance of PNT [9,10,11]. In addition, the abnormal condition of the satellite position would occur because of the impact of various perturbations while running the orbit. The earlier the maneuver is detected, the sooner the strategies of positioning and orbit determination can be adjusted [12,13,14,15,16,17,18]. The information on satellite maneuvers and anomalies is not available publicly, and are broadcasted on a time-interval basis (GPS: 2 h, BDS: 1 h). Therefore, the time of orbital maneuvers and anomalies are not accurate from the broadcast ephemeris, leading to a loss of many effective observations. Also, the errors in identification of abnormalities always appear in the Global Navigation Satellite System (GNSS) broadcast ephemeris, which decreases service performance in real-time. For these reasons, it is necessary to propose an optimized method to detect BDS orbit maneuvering and anomaly with higher time-resolved and reliability in real-time. At present, Yan and Ye et al. have detected the orbit maneuvering of the BeiDou GEO and IGSO using the mutual difference from the orbits before and after the maneuvering. Du et al. have determined the orbital maneuvers of the GEO satellites by the data of orbital monitor from the China Area Positioning System (CAPS); however, the data is unavailable for common users. In addition, the other GNSS constellations all belong to MEO satellites, and the maneuver times of MEO is few. Cui et al. determine the orbit maneuvering of the space objects using the orbital residual, but the mechanics model of the orbital maneuver was needed. Su et al. detected the orbital maneuver by using the mechanical energy difference between the spacecraft and space targets which could not be implemented in real-time. Although some research has been done for orbit maneuvering and anomaly [19,20,21,22,23,24,25,26], the real-time maneuver detection and determining the accurate time for orbit maneuvering are hardly realized by common users. In order to detect orbit maneuvering in real-time, we published a real-time detected method using Single Point Positioning (SPP) technology [27] which defines the satellite identification factor and the time discriminant factor to determine the PRN of the maneuverable satellite and the maneuver start time using the two-steps method. In the follow-up studies, we found that the SPP detected method has a delay (5–30 min) in detecting the orbital maneuver time because the precondition for detection results is the availability of positioning services. Furthermore, the previous paper did not involve the BDS MEO satellites, and the BDS orbital anomalies detection has not been studied deeply by Huang et al. in 2017.

For solving the above questions, we proposed a new one-step method with higher accuracy to simultaneously detect the PRN and the start time of the satellite orbit maneuvering. The classified non-maneuvering anomalies are detected by a new median robust method. The proposed method can be programmed as a data preprocessing tool to enhance the quality of GNSS services in the real-time applications, which not only benefits the reliability of real-time BeiDou but also that of other GNSS PNT services.

## 2. The Detection Theory of Orbital Maneuvers/Anomaly Using a One-Step Method

When the satellite is maneuvered or has an anomaly, the real-time satellite position calculated by the broadcast ephemeris is not correct given the increasing errors. The strategy of the BeiDou system is to mark the satellite health status as unhealthy about one hour before orbit maneuvering. This means that the real-time positions of satellite calculated by the broadcast ephemeris are not correct before about one hour, which reduces the utilization rate of available satellite data. On the other hand, the health marks for BDS satellites from the broadcast ephemeris are misidentified or sometimes missing, which leads to the information being received by common users to be unreliable. Considering that the real-time pseudo-range observations are not influenced by maneuvers or anomalies, the absolute residuals between pseudo-range observations and the pseudo-range calculated by the broadcast ephemeris will increase during the period of orbital maneuver or anomaly. Thus, a robust method using the pseudo-range residuals is proposed to detect the orbital maneuver and anomaly. This is an effective way that could be useful to common users on every static station.

The pseudo-range residual will contain increasing errors because of the satellite position being changed by orbit maneuvering. When the satellite orbit has an anomaly, the pseudo-range residual will change suddenly in the corresponding time period. These characteristics of different situations can be used for detection. Two factors—namely the orbital maneuver detection factor (*L_M_*) and the anomaly detection factor (S*_M_*)—were defined and used to detect the start time of orbit maneuvering and the period of non-maneuvering anomaly.

### 2.1. Orbital Maneuver Detection Factor

The orbital maneuver detection factor is calculated by subtracting the absolute residual value of the pseudo-range from the empirical threshold of the residual value of the pseudo-range. The modified pseudo-range (${\hat{D}}_{i}^{j}\left(k\right)$) is corrected for the receiver clock offsets, the ionospheric delay, and the tropospheric delay from the raw observations (${\tilde{D}}_{i}^{j}\left(k\right)$). ${\hat{D}}_{i}^{j}\left(k\right)$ is the credible variable to reflect the distance between the satellite and the station during the orbital maneuver period. The distance between the station and satellite is calculated using the broadcast ephemeris, which contains the gross error because of the unreliable orbital parameters provided by the broadcast ephemeris. $L_{i}^{j}\left(k\right)$ is the absolute residual value of observations which could be used to detect the orbit maneuvering and is computed by

(1) $$L_{i}^{j}\left(k\right)=\left|{\hat{D}}_{i}^{j}\left(k\right)-D_{i}^{j}\left(k\right)\right|$$

where | | is the function of the absolute value. ${\hat{D}}_{i}^{j}\left(k\right)$ is the modified pseudo-range. $D_{i}^{j}\left(k\right)$ is the distance calculated by the broadcast ephemeris and the coordinates of the station from the International GNSS Service (IGS). $L_{i}^{j}\left(k\right)$ is the absolute residual value of the pseudo-range. The orbital maneuver detection factor ($L_{M}^{j}\left(k\right)$) is defined and computed by

(2) $$L_{M}^{j}\left(k\right)=L_{i}^{j}\left(k\right)-L_{Max}^{j}$$

where the $L_{Max}^{j}$ is the empirical threshold of the pseudo-range observations belonging to satellite j. The empirical threshold $L_{Max}^{j}$ of satellite *j* is given in advance.

When $L_{M}^{j}\left(k\right)$ is bigger than 0 and keeps a continuous upward trend during a period (i.e., 10 min chosen in this paper), the corresponding time of $L_{M}^{j}\left(k\right)$ is considered to be the start moment of the orbit maneuver. For the empirical threshold of the observations for satellite j, $L_{Max}^{j}$ is the key to determine the start moment of orbit maneuvering. Considering that the residual of the observations obeys normal distribution, these residuals in the normal condition of the BDS satellite are all in the confidence interval of the 99.74% (3σ) confidence coefficient. In other words, if the factor is out of the corresponding confidence interval, it would be considered abnormal. Thus, the $L_{Max}^{j}$ for the BDS satellite is provided by the upper confidence interval of the residuals of the observations in the 99.74% confidence coefficient.

Because there is no uniform correction model of the receiver clock offset, the least-squares method can be used to estimate the receiver clock offset. It needs to be emphasized that the least-squares method would be evidently affected by the blunders. Once the orbital parameters in the broadcast ephemeris are unusable and have big biases, the receiver clock offset cannot be calculated correctly. Then, the pseudo-range observations corrected by this receiver clock offset will have big residuals compared with the calculated pseudo-range, even if the corresponding satellites are healthy and usable. This leads to a difficulty to confirm the PRN of the maneuvering satellite. Therefore, this paper proposed an optimized method of a robust weight matrix for the observations, in order to avoid disturbing the orbit deviation due to the pseudo-range residuals of normal satellites. This robust least-squares method is used to estimate the receiver clock offset as follows.

(3) $$V=B\delta t_{i}-l$$

(4) $$\delta t_{i}={\left(B^{T}\bar{P}B\right)}^{-1}B^{T}\bar{P}l$$

Equation (3) is the observation equation for estimating the receiver clock offset, where B is the coefficient matrix of the estimated parameters $\delta t_{i}$, l is a constant term, V is the corrections for observations, and $\bar{P}$ is the robust equivalence weight matrix.

Considering the rapid changes of $L_{i}^{j}\left(k\right)$ after the orbital maneuver or anomaly, the two-stage function of the robust equivalent weight matrix is structured as follows [27,28].

(5) $${\bar{p}}^{j}\left(k\right)=\{\begin{array}{cc} p^{j}\left(k\right) & \left|\frac{L_{i}^{j}\left(k\right)}{L_{Max}^{j}}\right|\leq r\\ 0 & \left|\frac{L_{i}^{j}\left(k\right)}{L_{Max}^{j}}\right|>r \end{array}$$

where the $p^{j}\left(k\right)$ is the diagonal element of the observation weight matrix for the *j* satellite at the *k* epoch, which is in accordance to the weight rule of the satellite elevation angle. ${\bar{p}}^{j}\left(k\right)$ is the diagonal element of robust equivalence weight matrix $\bar{P}$ and r is the critical index, which is assigned as 1.0 in this work.

If one satellite *j* is suspected to be the maneuvering orbit or non-maneuvering anomaly at the epoch *k*, ${\bar{p}}^{j}\left(k\right)$ would be assigned as 0 (i.e., 0 for doubtful maneuvering or anomaly, 1 for normal). Then the receiver clock offset at the next epoch *k* + 1 would be estimated with ${\bar{p}}^{j}\left(k\right)$ valued at 0. Thus, the $L_{i}^{j}\left(k\;+\;1\right)$ of other normal satellites assigned as 1 would not be influenced.

When $L_{i}^{j}\left(k\right)$ is bigger than 0 and shows a continuous growth trend within a time period, the first epoch with $L_{i}^{j}\left(k\right)>0$ is considered to be the start time of the maneuver.

### 2.2. Orbital Anomaly Detection Factor

When the satellite orbit has a non-maneuvering anomaly, the pseudo-range residual will change suddenly in the corresponding time period. If this change is too great, this leads to the increase of $L_{i}^{j}\left(k\right)$ for many satellites, and the ${\bar{p}}^{j}\left(k\right)$ of these satellites would be marked as 0. When $L_{i}^{j}\left(k\right)$ is more than 0, the satellite is considered a suspicious object for anomaly. The number of suspect objects is defined as Q in the same epoch. According to the magnitude of Q, the non-maneuvering anomaly is divided into two categories: weak anomaly and strong anomaly. When the value of Q is less than the redundant observation number, it is considered a weak anomaly. Otherwise, it is considered a strong anomaly. The redundant observation number for estimating the receiver clock offset is defined as R. R is equal to N−1, and N is the number of satellites in one epoch. The critical index r in Equation (5) is assigned as 3.0 in this work.

#### 2.2.1. The Weak Anomaly Detection Factor

The method to calculate the weak anomaly detection factor is the same as the method for the orbit maneuvering. The weak anomaly detection factor can be calculated by

(6) $$S_{M}^{j}{}^{-}\left(k\right)=L_{i}^{j}\left(k\right)-L_{Max}^{j}$$

where the $S_{M}^{j}{}^{-}\left(k\right)$ is the weak anomaly detection factor for *j* satellite at *k* epoch. When $S_{M}^{j}{}^{-}\left(k\right)$ is bigger than 0 and shows abnormal jumps in a time period, the corresponding period of $S_{M}^{j}{}^{-}\left(k\right)>0$ is considered to be the period of non-maneuvering weak anomaly.

#### 2.2.2. Strong Anomaly Detection Factor

When Q is greater than or equal to the redundant observation number (R), it is considered a strong anomaly. This will cause the receiver clock offset in the next epoch to have a low reliability or to not be solved. Thus, we used the median robust method to solve this problem as follows.

(7) $$\left[\begin{array}{c} V_{1}{}_{\;}\\ V_{2}\\ \vdots \\ V_{i} \end{array}\right]=B\left[\begin{array}{c} \delta t_{i}{}_{1}\\ \delta t_{i}{}_{2}\\ \vdots \\ \delta t_{i}{}_{N} \end{array}\right]-\left[\begin{array}{c} l_{1}\\ l_{2}\\ \vdots \\ l_{N} \end{array}\right]$$

(8) $$\delta t_{i}=median\left[\begin{array}{c} \delta t_{i}{}_{1}\\ \delta t_{i}{}_{2}\\ \vdots \\ \delta t_{i}{}_{N} \end{array}\right]$$

where V is the correction for observations of the satellite i. l is a constant term. *N* is the total number of observations.

${\hat{D}}_{i}^{j}\left(k\right)$ is corrected using $\delta t_{i}$ in Equation (8), then the new $L_{i}^{j}\left(k\right)$ and ${\bar{p}}^{j}\left(k\right)$ are updated using Equations (1)–(5). Then, the $\delta t_{i}$ will be calculated with the new ${\bar{p}}^{j}\left(k\right)$ using Equations (3) and (4). The following steps are the same with the steps in Section 2.1. The strong anomaly detection factor can be calculated by

(9) $$S_{M}^{j}{}^{+}\left(k\right)=L_{i}^{j}\left(k\right)-L_{Max}^{j}$$

where $S_{M}^{j}{}^{+}\left(k\right)$ is the factor of non-maneuvering strong anomaly for the *j* satellite at the *k* epoch. When $S_{M}^{j}{}^{+}\left(k\right)$ is bigger than 0 and shows abnormal jumps in a time period, the corresponding period of $S_{M}^{j}{}^{+}\left(k\right)>0$ is considered to be the period of the non-maneuvering strong anomaly. Specifically, when Q marked by the new ${\bar{p}}^{j}\left(k\right)$ is equal to N, it is considered the anomaly for the user terminal.

### 2.3. Steps for Orbital Maneuver and Anomaly Detection

The following steps are used for orbital maneuver detection in this work.

(1) The $L_{M}^{j}\left(k\right)$ is calculated using Equations (1)–(4).
(2) If the $L_{M}^{j}\left(k\right)$ is greater than 0, ${\bar{p}}^{j}\left(k\right)$ is calculated using Equation (5).
(3) The $L_{M}^{j}\left(k+1\right)$ for the next epoch is calculated using Equations (1)–(4) with ${\bar{p}}^{j}\left(k\right)$. Repeat the steps (2) and (3).
(4) If $L_{M}^{j}\left(k\right)$ keeps a continuous growth trend in M epochs, the first epoch with $L_{M}^{j}\left(k\right)$ greater than 0 is considered to be the start time of the orbit maneuver. M is assigned as 20 in this study.

The following steps are used for orbital anomaly detection in this work.

(1) The $S_{M}^{j}\;\left(k\right)$ is calculated by Equations (1)–(4).
(2) If the $S_{M}^{j}\;\left(k\right)$ is greater than 0, ${\bar{p}}^{j}\left(k\right)$ and Q are calculated by Equation (5).
(3) Compare the Q with the R. If Q is smaller than R, go to step (4); otherwise, go to step (5).
(4) The value of $S_{M}^{j}{}^{-}\left(k\right)$ is assigned by $S_{M}^{j}\;\left(k\right)$. Then, we go to the next epoch k + 1.
(5) $\delta t_{i}$ is calculated using Equations (7) and (8), and ${\hat{D}}_{i}^{j}\left(k\right)$ is corrected using $\delta t_{i}$ in Equation (8). Then, the new $L_{i}^{j}\left(k\right)$ and ${\bar{p}}^{j}\left(k\right)$ are updated. The new $S_{M}^{j}\;\left(k\right)$ will be calculated with the new ${\bar{p}}^{j}\left(k\right)$ using Equations (1–4). The value of $S_{M}^{j}{}^{+}\left(k\right)$ is assigned using $S_{M}^{j}\;\left(k\right)$.
(6) The $S_{M}^{j}\;\left(k+1\right)$ for the next epoch is calculated using Equations (1)–(4) with ${\bar{p}}^{j}\left(k\right)$.
(7) This applies if $S_{M}^{j}{}^{+}\left(k\right)/S_{M}^{j}{}^{-}\left(k\right)$ shows abnormal jumps in M epochs. M is assigned as 20 in this study. The period where $S_{M}^{j}{}^{+}\left(k\right)/S_{M}^{j}{}^{-}\left(k\right)$ is greater than 0 is considered the period of orbital anomaly.

When the Q marked by the new ${\bar{p}}^{j}\left(k\right)$ is equal to N, it is considered as the anomaly for user terminal.

## 3. Numerical Examples

### 3.1. Data Description

**Figure 1:**
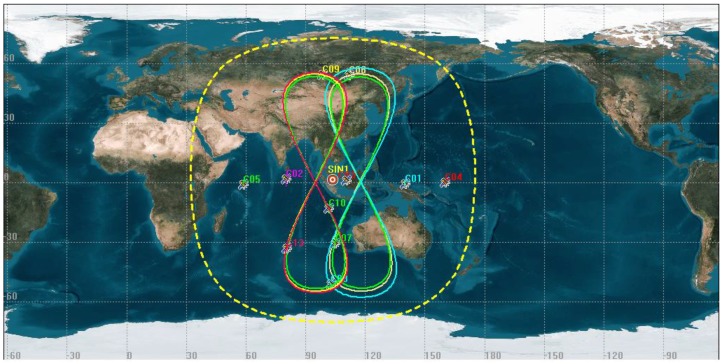
The location of the selected station and the distributions of the trajectories for Geostationary Orbit (GEO)/Inclined Geosynchronous Orbit (IGSO) satellites.

The data of the Multi-GNSS Experiment (MGEX) station SIN1 was selected to analyze the detection results of BeiDou GEO and IGSO satellites’ orbital maneuvers and anomalies. The station SIN1 is located in Singapore (1°20′ N, 103°40′ E, see Figure 1) with an LEIAR25.R3 GNSS receiver. The sample interval is 30 s. The coordinates of SIN1 can be achieved from the IGS Solution Independent Exchange (SINEX) product or using the Precise Point Positioning technique. In this work, the SINEX coordinates of SIN1 are chosen to verify the detection performance of GEO and IGSO. The distributions of trajectories on the station SIN1 are shown in Figure 1.

From Figure 1, the locations of the SIN1 station are marked as red circles. The satellites moving with trajectories in “8” shapes are IGSO satellites, and the satellites with trajectories in a point shape are GEO satellites. The satellites with distributions of trajectories with in the yellow circle can be observed by SIN1.

The thresholds of the residuals of the pseudo-range for different satellites of the SIN1 station on doy (day of year) 029 (29 January), doy 140 (20 May), doy 229 (17 August), and doy 296 (23 October) of 2017 are shown in Table 1.

The blue marked PRNs are the GEO satellites, and the unmarked PRNs are the IGSO satellites. Consider that the thresholds of the residuals of the pseudo-range keep steady only for a short period of time, which is not over one week [27]. Table 1 shows that the values of $L_{Max}$ are different in different days. Therefore, $L_{Max}$ should be updated frequently, and we suggest updating it every three days.

**Figure 2:**
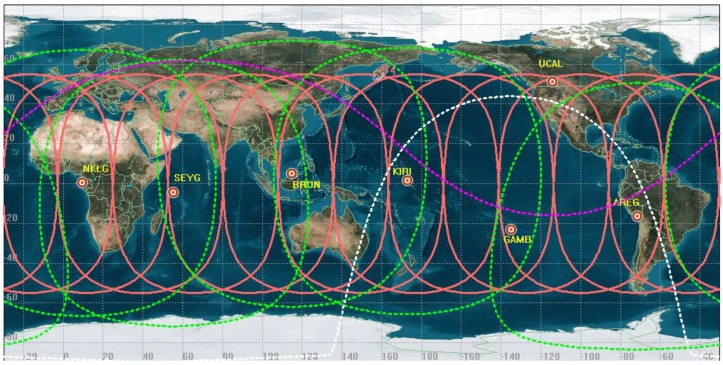
The location of the selected stations and the distributions of trajectories for MEO satellites.

In addition, the selected stations for maneuver detection of BDS MEO satellites are show in Table 2. The distributions of trajectories on the ground and the location of the selected stations are shown in Figure 2.

In Figure 2, the locations of the selected stations are marked as red circles. The red trajectory is for a MEO satellite on the ground during a regression period. The area of green circle is the operating range of MEO satellites monitored by the corresponding station, which is analyzed by the Systems Tool Kit (STK). The area above the purple line is the range monitored by the UCAL station (The station UCAL is located in Canada with the TRIMBLE NETR9 GNSS receiver), and the area under the white line is the range monitored by GAMB station (The station GAMB is located in French Polynesia with the TRIMBLE NETR9 GNSS receiver). The seven selected stations using the proposed method ensure that the operating range of MEO satellites can be monitored for orbital maneuver and anomaly detection all the time.

### 3.2. The Orbital Maneuver Detection for BDS

**Figure 3:**
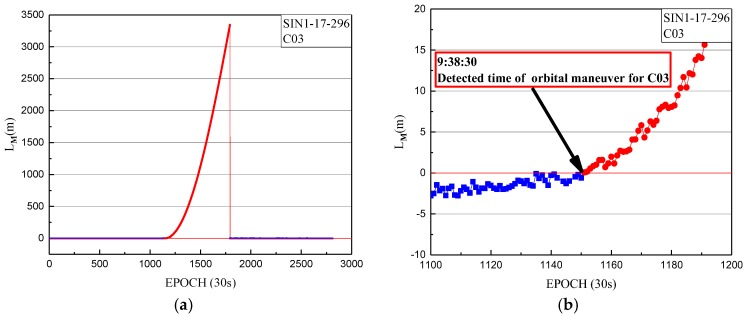
(**a**) The orbital maneuver detection factor of SIN1 station for C03 on 23 October 2017 from epoch 0 to 2880. (**b**) The orbital maneuver detection factor of SIN1 station for C03 on 23 October 2017 from epoch 1100 to 1200, with a smaller range for the vertical axis. The orbital maneuver detection factors of less than 0 are indicated by blue dots. The orbital maneuver detection factors of more than 0 are indicated by red dots.

Using the one-step method, the orbital maneuver was detected for C03 on 23 October 2017. The detection results of orbit maneuvering for SIN1 are firstly given, with the orbital maneuver detection factor series shown in Figure 3.

In Figure 3, the detection factor series shows a trend continually on the rise of about 4.5 h. The value of the orbital maneuver detection factor is greater than 0 from 9:38:30. The start time of the orbit maneuvering of C03, determined by the orbital maneuver detection factor on 23 October 2017, is 9:38:30.

We used the health marks for satellites (i.e., 0 for healthy status and 1 for unhealthy status) from the broadcast ephemeris provided by MGEX and the precise orbit products from international GNSS Monitoring & Assessment System (iGMAS) to verify the correction of the orbit maneuvering detection results.

**Figure 4:**
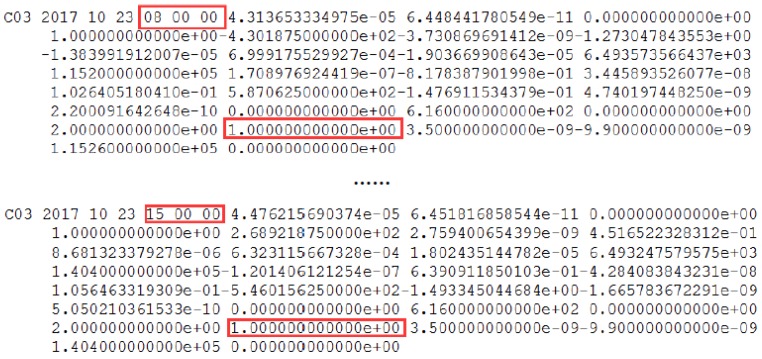
The C03 satellite is marked unhealthy in the broadcast ephemeris on 23 October 2017. The red square marks the unhealthy status and the corresponding time. C03 is unhealthy and is marked as 1 between 9:00:00 and 14:00:00, which is not shown because of the reasonable length limitation.

**Figure 5:**
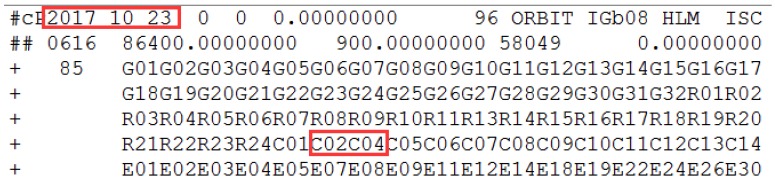
The information of the header file from the precise orbit products provided by iGMAS on 23 October 2017. The red squares mark the corresponding date and highlight the precise orbit of C03 cannot be determined.

The health marks for satellites in the broadcast ephemeris of C03 and the header information of the precise orbit products are shown in Figure 4 and Figure 5.

In Figure 4, the health marks for the satellites from the broadcast ephemeris could be marked unhealthy ahead of orbit maneuvering. Specifically, C03 is marked unhealthy from 8:00:00, while it is detected as orbit maneuvering at 9:38:30. Satellite orbit cannot be precisely determined by the conventional model of orbital mechanics due to the intervention of the orbiting maneuvering force. The C03 satellite misses based on the header of the precise orbit products for the corresponding date, indicating the correction of orbit maneuvering detection results.

**Figure 6:**
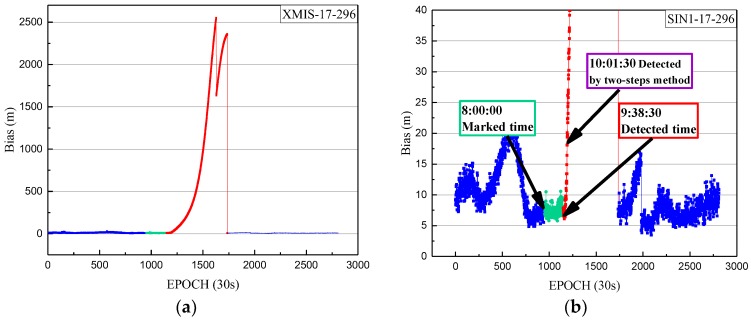
(**a**) The deviations of Single Point Positioning (SPP) on doy 296 of 2017 from epoch 0 to 2880; (**b**) The deviations of SPP with a smaller range for the vertical axis. The deviations of SPP results in three-dimensional space corresponding to the healthy period in the broadcast ephemeris are indicated by the blue dots. The SPP deviations corresponding to the unhealthy period in the broadcast ephemeris with an orbital maneuver detection factor of less than 0 are indicated by green dots. The SPP deviations corresponding to the unhealthy period in the broadcast ephemeris after the detected orbital maneuver start time are indicated by red dots.

Also, the SPP results of another site named XMIS (located in Christmas Island, AU 10° 26′ S, 105° 41′ E) were used to further validate the correction of the detection results. The SPP deviations of XMIS on doy 296 of 2017 are shown in Figure 6.

In Figure 6, the detected start time of orbit maneuvering and the start time of the unhealthy marks are pointed out. The deviations marked blue and green are in same level, while the deviations marked red are obviously higher and show a rapid upward trend. Thus, the satellite starts to maneuver at 1–1.5 h after it is marked unhealthy in the broadcast ephemeris. This strategy results in the loss of observations for 1–1.5 h and reduces the utilization of valid observations. On the other hand, after the detected start time of orbit maneuvering, the SPP deviation gradually increases to a tendency where the positioning result is not reliable. The above positioning results can further prove the correction of the detected start time of orbit maneuvering. In order to justify that the start time of orbital maneuver detected by this method is more accurate than previous research (Huang et al., 2017), the start time of orbit maneuvering for C03 on 23 October 2017 (which calculated by the previous method) is 10:01:30. There is a delay of about 23 min from the start time detected by the two-steps method.

The applicability of the optimized method for detecting the orbit maneuvering of IGSO and MEO was verified by the detected results, which are not shown in this paper because of the reasonable length limitation.

### 3.3. The Orbital Maneuver Detection for GPS

**Figure 7:**
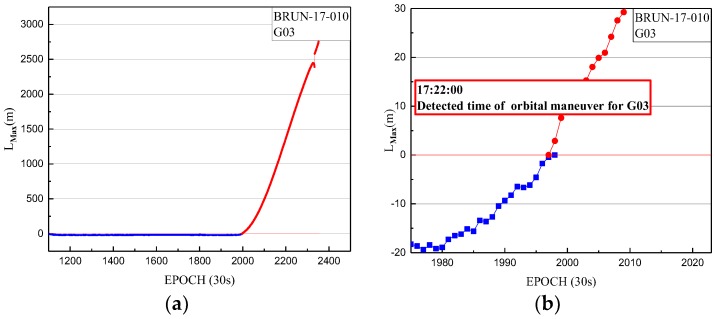
(**a**) The orbital maneuver detection factor of BRUN station for G03 on 10 January 2017 from epoch 1102 to 2355; (**b**) The orbital maneuver detection factor of BRUN station for G03 on 10 January 2017 from epoch 1975 to 2025, with a smaller range for the vertical axis. The orbital maneuver detection factors of less than 0 are indicated by blue dots. The orbital maneuver detection factors of more than 0 are indicated by red dots.

In order to verify the applicability of the detection method for orbital maneuvers in other GNSS MEO constellations using the one-step method, the orbital maneuver is detected for G03 satellite of GPS on 10 January 2017. The orbit maneuvering has been detected by the BRUN station. The results of BRUN are shown, with the orbital maneuver detection factor series shown in Figure 7.

In Figure 7, the detection factor series shows a continually rising trend for hours. The value of the orbital maneuver detection factor is greater than 0 from 17:22:00. The start time of the orbit maneuvering of G03 is determined by the orbital maneuver detection factor on 10 January 2017, and is 17:22:00.

We used the health status marks for satellites (i.e., 0 for healthy status and 1 for unhealthy status) from the broadcast ephemeris provided by MGEX and the precise orbit products from iGMAS to verify the correction of the orbit maneuvering detection results. This is not shown because of the reasonable length limitation. The health status marks of satellite G03 in the broadcast ephemeris is marked as unhealthy from 15:59:44 to 21:57:52. In addition, the satellites’ orbit cannot be precisely determined using the conventional model of orbital mechanics due to the intervention of the orbiting maneuvering force. The G03 satellite misses based on the header of the precise orbit products from iGMAS for the corresponding date, indicating the correction of orbit maneuvering detection results. Therefore, the new one-step method also can be used to determine the start time of orbital maneuvers of other GNSS MEO constellations.

### 3.4. The Non-Maneuvering Anomaly Detection

#### 3.4.1. Non-Maneuvering Anomaly Detection for a Weak Anomaly

**Figure 8:**
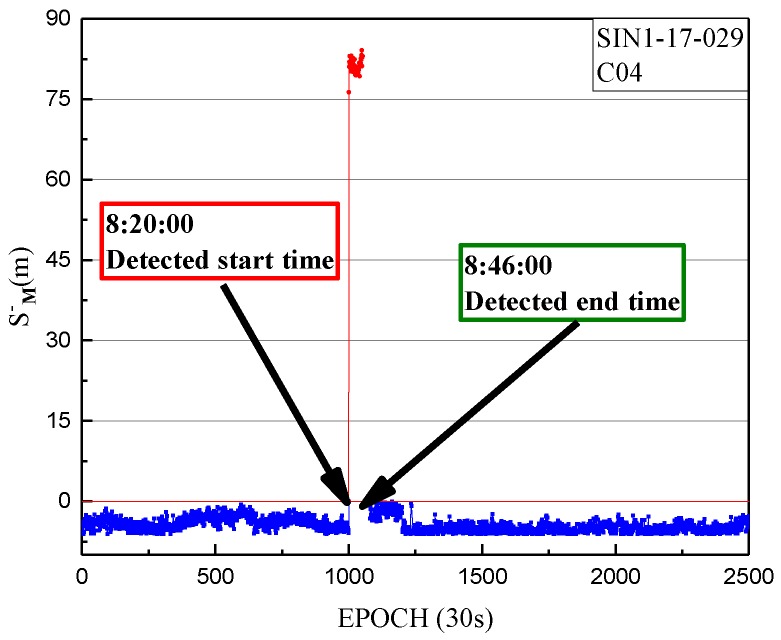
The weak anomaly detection factor of SIN1 station for C04 on 29 January 2017. The weak anomaly detection factors of less than 0 are indicated by blue dots. The weak anomaly detection factors of more than 0 are indicated by red dots.

The non-maneuvering anomaly was detected as a weak anomaly on 29 January 2017. The performance of the non-maneuvering weak anomaly detection for SIN1 is firstly given, with an anomaly detection factor series shown in Figure 8.

In Figure 8, the detection factor series shows that the period of non-maneuvering anomaly for C04 detected by the weak anomaly detection factor is 8:20:00 to 8:46:00 on 29 January 2017.

In order to justify the correction of the detection results for SIN1, the precise orbit products provided by iGMAS and the pseudo-range SPP bias of the other station, XMIS, are used as references.

We used the information of precise orbit products from iGMAS to verify the correction of the orbital anomaly detection results. The pseudo-range SPP bias of the other station, XMIS, was used as a reference.

**Figure 9:**
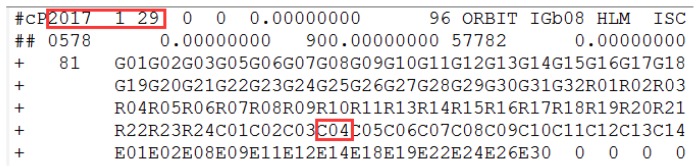
The information of the header file from the precise orbit products on 29 January 2017. The red squares mark the corresponding date and highlight the precise orbit of C04 has been determined.

The information of the header file from the precise orbit products provided by iGMAS contained the C04 satellite in Figure 9. It is proved that there was no orbital maneuver for the C04 satellite.

**Figure 10:**
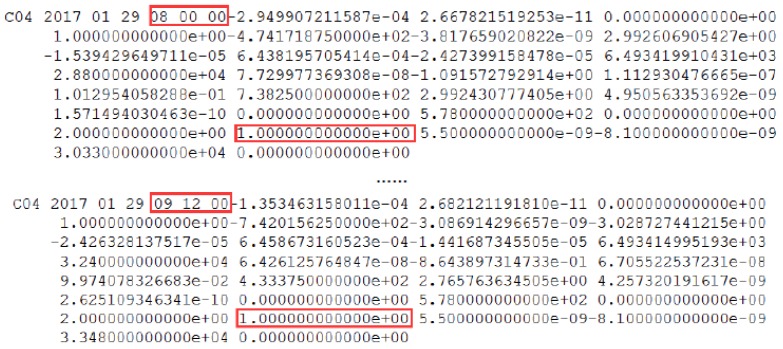
The C04 satellite is marked unhealthy in the broadcast ephemeris on 29 January 2017. The red square marks the unhealthy status and the corresponding time. C04 is unhealthy and is marked as 1 between 8:00:00 and 9:12:00, which is not shown because of the reasonable length limitation.

**Figure 11:**
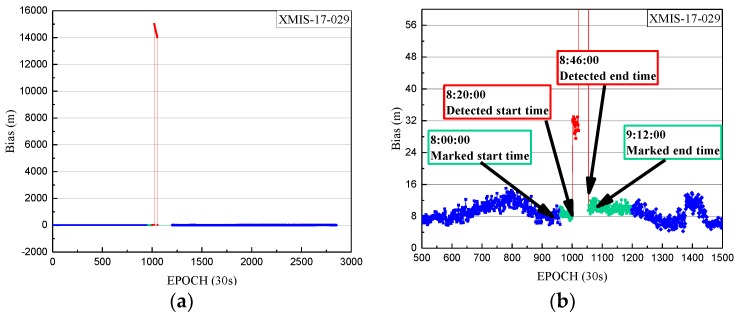
(**a**) The deviations of SPP on doy 029 of 2017 from epoch 0 to 2880. (**b**) The deviations of SPP on doy 029 of 2017 from epoch 500 to 1500, with a smaller range for the vertical axis. The deviations of SPP results in three-dimensional space corresponding to the healthy period in the broadcast ephemeris are indicated by the blue dots. The SPP deviations corresponding to the unhealthy period in the broadcast ephemeris with a weak anomaly detection factor of less than 0 are indicated by green dots. The SPP deviations corresponding to the unhealthy period in the broadcast ephemeris during the detected period are indicated by red dots.

Also, the health marks of C04 and the SPP results of another site, XMIS, is used to further validate the correction of the detection results. The SPP deviations of XMIS on doy 029 of 2017 is shown in Figure 10 and Figure 11.

In Figure 11, the detected periods of non-maneuvering anomaly and the periods of unhealthy marks are pointed out. The deviations marked blue and green are in same level, while the deviations marked red are obviously higher and show a jumping trend. The deviations of SPP results between the marked period and the detected period are usable and not influenced by the non-maneuvering anomaly. Figure 11 can verify that the usable data is lost, with the unhealthy status marked in the broadcast ephemeris; the detection method can improve the utilization rate of the observed data. In addition, in Figure 11, the deviations after the detected period jumped sharply due to the non-maneuvering anomaly, rendering the SPP results unreliable. Also, this period (from 8:20:00 to 8:46:00) is definitely close to the real period of the non-maneuvering anomaly.

#### 3.4.2. Non-Maneuvering Anomaly Detection for a Strong Anomaly

**Figure 12:**
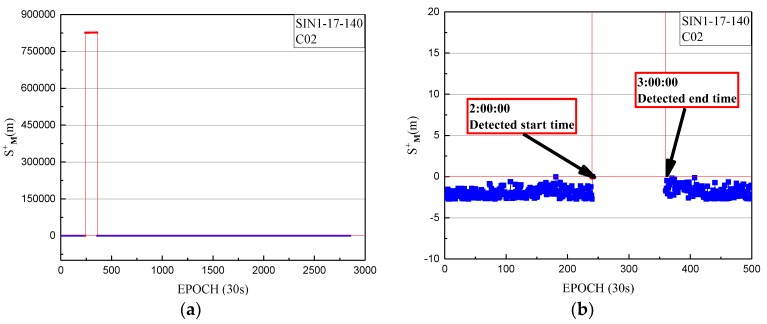
(**a**) The non-maneuvering strong anomaly detection factor of SIN1 station for C02 on 20 May 2017 from epoch 0 to 2880; (**b**) The non-maneuvering strong anomaly detection factor of SIN1 station for C02 on 20 May 2017 from epoch 0 to 500, with a smaller range for the vertical axis. The strong anomaly detection factors of less than 0 are indicated by blue dots. The strong anomaly detection factors of more than 0 are indicated by red dots.

The non-maneuvering anomaly was detected as a strong anomaly on 20 May 2017. The performance of the non-maneuvering strong anomaly detection for SIN1 is given, with the non-maneuvering strong anomaly detection factor series shown in Figure 12.

In Figure 12, the detection factor series shows that the period of non-maneuvering anomaly for C02 detected by the strong anomaly detection factor is 2:00:00 to 3:00:00 on 20 May 2017.

**Figure 13:**
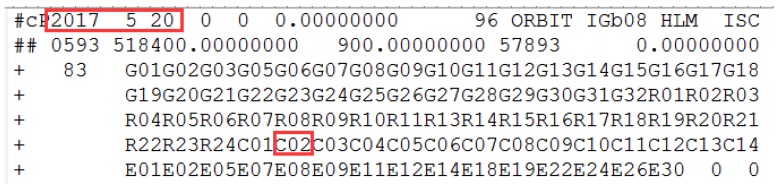
The information of header file from the precise orbit products on 20 May 2017. The red squares mark the corresponding date and highlight the precise orbit of C02 has been determined.

The information of precise orbit products from iGMAS is as shown in Figure 13. The SPP deviations of the other station, XMIS, are used as references.

The information of the header file from the precise orbit products provided by iGMAS contained the C02 satellite in Figure 13. It is proven that there is no orbit maneuvering for the C02 satellite.

**Figure 14:**
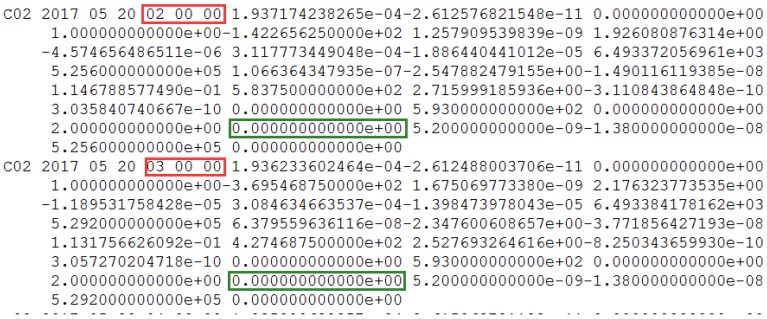
The C02 satellite is marked healthy in the broadcast ephemeris on 20 May 2017. The green square marks the healthy status and the red square marks the corresponding time. C02 is healthy and is marked as 1 between 2:00:00 and 3:00:00.

**Figure 15:**
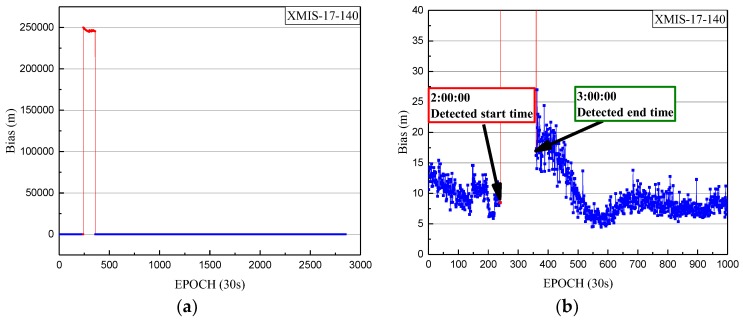
(**a**) The deviations of SPP on doy 140 of 2017 from epoch 0 to 2880; (**b**) The deviations of SPP on doy 140 of 2017 from epoch 0 to 1000, with a smaller range for the vertical axis. The deviations of SPP results in three-dimensional space corresponding to the healthy period in broadcast ephemeris are indicated by the blue dots. The SPP deviations corresponding to the detected period are indicated by red dots.

Also, the health marks of C02 and the SPP results of another site, XMIS, is used to further validate the correction of the detection results. The SPP deviations of XMIS on doy 140 of 2017 is shown in Figure 14 and Figure 15.

In Figure 15, the detected periods of non-maneuvering anomalies are pointed out. The SPP deviations after the detected period jumped sharply because of the non-maneuvering anomaly, leading the SPP results to be unreliable. Also, this period (from 2:00:00 to 3:00:00) is definitely close to the real period of the non-maneuvering anomaly. From Figure 14, the healthy status of C02 is marked as 0 between 2:00:00 and 3:00:00 in the broadcast ephemeris. Therefore, the real-time robust detection method not only improves the utilization of observations for common users with effective data, but also makes an effective supplement for the health status of the BeiDou satellites in the broadcast ephemeris.

### 3.5. Orbit Maneuvering and Anomaly Detection for the BeiDou Satellites in 2017

We used the results from 001 to 300 doy of 2017 to verify the applicability of the optimized method for long-term orbit maneuvering and anomaly detection. The detected results are compared with the health marks from the broadcast ephemeris. The detected orbit maneuverings are all marked unhealthy in the period. The results are shown in Table 3. For the non-maneuvering anomaly, some health marks in the broadcast ephemeris are missed for BDS satellites, as shown in Table 4.

Table 3 shows that there were 46 orbital maneuvers of BDS satellites in 2017 (001 to 300 doy). IGSO satellites had 13 orbital maneuvers. MEO satellites had only one orbital maneuver. This is because the GEO satellites need more maneuvers to maintain the static character with Earth because of weak observation geometry and the lack of orbit dynamics [29]. The frequency of orbit maneuvering for GEO satellites is higher than that for IGSO and MEO satellites. The average time difference between the detected start moment using the method and the marked started moment from the broadcast ephemeris for BeiDou satellites is 91 min, which is the time period the data is still available in. Therefore, the optimized method could detect orbit maneuvering more accurately in real-time and improve the utilization of observations.

Table 4 shows that the BeiDou GEO satellites had 102 non-maneuvering anomalies in year 2017. IGSO satellites had 13 non-maneuvering anomalies. MEO satellites had 12 non-maneuvering anomalies. The frequency of non-maneuvering anomalies for GEO satellites is higher than that for IGSO and MEO satellites. The average duration in difference between the marked period and the detected period is 95 min, which is the time period when the observational data is available. There were 17 unmarked health marks for BDS in the broadcast ephemeris. Therefore, the proposed detection method for an orbital anomaly not only improves the utilization of observations, but also supplements the information of health status for satellites in the broadcast ephemeris.

Orbit maneuvering and non-maneuvering anomalies appeared in the same day (C01 and C04 on 9 January 2017). This can be detected by the orbital maneuver detection factor and the anomaly detection factor in real-time. Thus, the proposed method performs well even when the orbital maneuvers and non-maneuvering anomalies appear on the same day.

## 4. Discussion and Conclusions

In this paper, an optimized robust method is proposed to detect orbit maneuvering and non-maneuvering anomalies for BeiDou GEO, IGSO, and MEO satellites using only the pseudo-range observations, the broadcast ephemeris, and the known station coordinates. The data of MGEX are analyzed, and the results show that the satellite orbital maneuvers and the non-maneuvering anomalies can both be detected accurately. In addition, the average time of orbit maneuvering differences between the marked started time in the broadcast ephemeris and the detected start time was 91 min in 2017. It indicates that the presented detection method for orbit maneuvering can enlarge the available observational data by about 1.52 h when the satellite maneuvers. The average duration of the non-maneuvering anomaly difference between the marked period of the broadcast ephemeris and the detected period was 94.95 min in 2017, which indicates that the presented detection method for orbital anomaly could enlarge the available observational data by about 1.58 h when the satellite has an anomaly. The proposed method of orbit maneuvering detection and anomaly detection could be used well together in real-time.

Also, the presented method is more effective and could be implemented more easily in any BeiDou static station. The proposed method could improve the PNT service during orbit maneuvers and anomalies; in addition, it also can be programmed as a data preprocessing tool to enhance the quality and reliability of GNSS real-time services in the applications.

## Figures and Tables

**Table 1:** The thresholds of the residuals of the pseudo-range (unit: meter).

	29 January 2017	20 May 2017	17 August 2017	23 October 2017
	$L_{Max}$			
C01	3.086	3.140	2.922	3.272
C02	3.082	2.718	2.950	3.463
C03	2.543	2.333	3.206	2.825
C04	3.733	3.658	4.294	3.601
C05	4.432	2.861	3.383	3.414
C06	4.660	6.334	7.298	5.288
C07	4.982	6.240	7.117	6.197
C08	4.322	4.244	5.130	4.965
C09	5.133	4.830	5.969	4.859
C10	5.268	5.761	5.570	5.515
C13	4.779	4.384	5.040	5.575

**Table 2:** The selected stations for Medium Earth Orbit (MEO) maneuver detection.

Station	Lat	Long	City	Country	Receiver
NKLG	0.35°	9.67°	Libreville	Gabon	TRIMBLE NETR9
SEYG	−4.68°	55.53°	Pointe Larue	Seychelles	TRIMBLE NETR9
BRUN	4.97°	114.95°	Gadong	Brunei	TRIMBLE NETR9
KIRI	1.35°	172.92°	Betio	Kiribati	TRIMBLE NETR9
GAMB	−23.13°	−134.97°	Rikitea	French Polynesia	TRIMBLE NETR9
UCAL	51.08°	−114.13°	Calgary	Canada	TRIMBLE NETR9
AREG	−16.47°	−71.49°	Arequipa	Peru	TRIMBLE NETR9

**Table 3:** Results of orbit maneuvering detection in 2017 for BDS (measure in hour: minute: second). The detected satellites, the times of orbital maneuvers, and the average time differences between the detected start moment and the marked moment are shown.

PRN	Times of Orbital Maneuvers	Average Time Differences	PRN	Times of Orbital Maneuvers	Average Time Differences
C01	10	1:30:36	C07	2	1:02:45
C02	8	1:32:52	C08	2	0:40:15
C03	12	1:56:42	C09	2	0:33:00
C04	4	1:09:00	C10	1	0:44:00
C05	12	0:54:33	C13	2	0:52:00
C06	4	1:36:00	C11	1	12:21:00
Average time differences between marked and detected				**1:31:05**	

**Table 4:** The detection results for non-maneuvering anomalies in 2017 for BeiDou satellites (measured in hour: minute: second). The detected satellites, the times of anomalies, and the average duration differences between the marked period and the detected period are shown.

PRN	Times of Anomalies	Average Duration of Differences	PRN	Times of Anomalies	Average Duration of Differences
C01	1	0:13:30	C08	3	0:17:30
C02	13	1:59:03	C09	1	Unmarked
C03	35	0:37:50	C10	1	1:15:30
C04	39	1:02:49	C13	3	0:03:00
C05	14	0:48:09	C11	6	11:08:30
C06	4	0:13:15	C12	3	Unmarked
C07	1	1:29:30	C14	3	6:40:15
The total anomaly number			**127**	Average different duration	**1:34:57**

## References

[1] Yang Y., Li J., Xu J., Tang J., Guo H., He H. (2011). Contribution of the compass satellite navigation system to global PNT users. Chin. Sci. Bull..

[2] Li X., Ge M., Dai X., Ren X., Fritsche M., Wickert J., Schuh H. (2015). Accuracy and reliability of multi-GNSS real-time precise positioning: GPS, GLONASS, BeiDou, and Galileo. J. Geod..

[3] Montenbruck O., Hauschild A., Steigenberger P., Hugentobler U., Teunissen P., Nakamura S. (2013). Initial assessment of the COMPASS/BeiDou-2 regional navigation satellite system. GPS Solut..

[4] Tu R., Lu C., Zhang P., Zhang R., Liu J., Lu X. (2017). The study of BDS RTK algorithm based on zero-combined observations and ionosphere constraints. Adv. Space Res..

[5] Zhang R., Zhang Q., Huang G., Wang L., Qu W. (2015). Impact of tracking station distribution structure on BeiDou satellite orbit determination. Adv. Space Res..

[6] Ge H., Li B., Ge M., Shen Y., Harald S. (2017). Improving BeiDou precise orbit determination using observations of onboard MEO satellite receivers. J. Geod..

[7] Hofmann-Wellenhof B., Lichtenegger H., Wasle E. (2008). GNSS-Global Navigation Satellite Systems: GPS, GLONASS, Galileo and More.

[8] Chen Z.G., Shuai P., Qu G. (2009). Analysis of the technical character and develop tendency of modern satellite navigation system. Sci. China Ser. E Techol. Sci..

[9] Wang B., Lou Y., Liu J., Zhao Q., Su X. (2016). Analysis of BDS satellite clocks in orbit. GPS Solut..

[10] Shi C., Zhao Q., Hu Z., Liu J. (2013). Precise relative positioning using real tracking and IGSO satellites. GPS Solut..

[11] Cao F., Yang X., Li Z., Sun B., Kong Y., Chen L., Feng C. (2014). Orbit determination and prediction of GEO satellite of BeiDou during repositioning maneuver. Adv. Space Res..

[12] Zhao Q., Wang C., Guo J., Liu X. (2015). Assessment of the Contribution of BeiDou GEO, IGSO, and MEO Satellites to PPP in Asia-Pacific Region. Sensors.

[13] Liu T., Yuan Y., Zhang B., Wang N., Tan B., Chen Y. (2016). Multi-GNSS precise point positioning (MGPPP) using raw observations. J. Geod..

[14] Steigenberger P., Hugentobler U., Hauschild A., Montenbruck O. (2013). Orbit and clock analysis of Compass GEO and IGSO satellites. J. Geod..

[15] Geng T., Su X., Fang R., Xie X., Zhao Q., Liu J. (2016). BDS Precise Point Positioning for Seismic Displacements Monitoring: Benefit from the High-Rate Satellite Clock Corrections. Sensors.

[16] Byun S.H. (2003). Satellite orbit determination using triple-differenced GPS carrier phase in pure kinematic mode. J. Geod..

[17] Guo R., Chen J., Zhu L., Tang G., Wu X. (2017). Kinematic Orbit Determination Method Optimization and Test Analysis for BDS Satellites with Short-arc Tracking Data. Acta Geod. Cartogr. Sin..

[18] Li Z., Zhang W., Gong X., Qu X. (2011). Solution of orbit maneuver problem in autonomous orbit determination of navigation satellites. Geomat. Inf. Sci. Wuhan Univ..

[19] Cui H., Liu W., Tang G., Song B., Ge M. (2016). Different Thrust Maneuvers Detection of Uncooperative Space Objects. J. Astronaut..

[20] Yan X., Huang G., Zhang R., Zhang Q. (2015). A Method Based on Broadcast Ephemeris to Detect BDS Satellite Orbital Maneuver. J. Navig. Position.

[21] Ye F., Yuan Y., Tan B., Jikun O. (2017). A Robust Method to Detect BeiDou Navigation Satellite System Orbit Maneuvering/Anomalies and Its Applications to Precise Orbit Determination. Sensors.

[22] Su J., Dong Y. (2012). Detection of space target orbit maneuver on board by wavelet analysis. Chin. J. Space Sci..

[23] Du L., Zhang Z., Li X., Wang R., Liu L., Guo R. (2014). Station-keeping Maneuver Monitoring and Moving-Window Ground Track Fitting of GEO Satellites. Acta Geod. Cartogr. Sin..

[24] Song W., Wang R., Wang J. (2012). A simple and valid analysis method for orbit anomaly detection. Adv. Space Res..

[25] Ha J., Heo M., Nam G. Anomaly Detection of IGS Predicted Orbits for Improvement of Near-Real-Time Positioning Accuracy Using GPS. Proceedings of the EGU General Assembly 2013.

[26] Liu Y., Chang Z., He F., Guo R. (2010). GEO Satellite Abnormity Recognition Based on Wavelet Analysis. J. Geod. Geodyn..

[27] Huang G., Qin Z., Zhang Q., Wang L., Yan X., Fan L., Wang X. (2017). A Real-Time Robust Method to Detect BeiDou GEO/IGSO Orbital Maneuvers. Sensors.

[28] Yang Y., Song L., Xu T. (2002). Robust estimator for correlated observations based on bifactor equivalent weights. J. Geod..

[29] Deng Z., Ge M., Uhlemann M., Zhao Q. Precise orbit determination of Beidou Satellites at GFZ. Proceedings of the EGU General Assembly Conference, IGS Workshop.

